# *Enterococcus faecalis*-Induced Macrophage Necroptosis Promotes Refractory Apical Periodontitis

**DOI:** 10.1128/spectrum.01045-22

**Published:** 2022-06-16

**Authors:** Xingzhu Dai, Rongyang Ma, Weiyi Jiang, Zilong Deng, Lijuan Chen, Yuee Liang, Longquan Shao, Wanghong Zhao

**Affiliations:** a Department of Stomatology, Nanfang Hospital, Southern Medical University, Guangzhou, China; b Stomatology Hospital, Southern Medical University, Guangzhou, China; University of Florida

**Keywords:** *Enterococcus faecalis*, necroptosis, refractory apical periodontitis, inflammation, bone loss, innate immunity

## Abstract

The persistence of residual bacteria, particularly Enterococcus faecalis, contributes to refractory periapical periodontitis, which still lacks effective therapy. The role of receptor-interacting protein kinase 3 (RIPK3)- and mixed lineage kinase domain-like protein (MLKL)-mediated necroptosis, a highly proinflammatory form of regulated cell death, has recently drawn much attention. However, the role of necroptosis in the pathogenesis of refractory periapical periodontitis remains unclear. We investigated whether the RIPK3/MLKL signaling pathway was activated in periapical lesion specimens obtained from patients diagnosed with refractory periapical periodontitis. *RIPK3*-deficient mice were then used to determine the role of necroptosis under this condition *in vivo*. We found that the phosphorylation levels of RIPK3 and MLKL were elevated in periapical lesion specimens of patients with refractory periapical periodontitis. In addition, necroptosis was induced in an E. faecalis-infected refractory periapical periodontitis mouse model, in which inhibition of necroptosis by RIPK3 deficiency could markedly alleviate inflammation and bone destruction. Moreover, double-labeling immunofluorescence suggested that macrophage necroptosis may be involved in the development of refractory periapical periodontitis. Then, we established an *in vitro* macrophage infection model with E. faecalis. E. faecalis infection was found to induce necroptotic cell death in macrophages through the RIPK3/MLKL signaling pathway, which was markedly alleviated by the RIPK3- or MLKL-specific inhibitor. Our study revealed that RIPK3/MLKL-mediated macrophage necroptosis contributes to the development of refractory periapical periodontitis and suggests that inhibitors or treatments targeting necroptosis represent a plausible strategy for the management of refractory periapical periodontitis.

**IMPORTANCE** Oral infectious diseases represent a major neglected global population health challenge, imposing an increasing burden on public health and economy. Refractory apical periodontitis (RAP), mainly caused by Enterococcus faecalis, is a representative oral infectious disease with considerable therapeutic challenges. The interplay between E. faecalis and the host often leads to the activation of programmed cell death. This study identifies an important role of macrophage necroptosis induced by E. faecalis in the pathogenesis of RAP. Manipulating RIPK3/MLKL-mediated necroptosis may represent novel therapeutic targets, not only for RAP but also for other E. faecalis-associated infectious diseases.

## INTRODUCTION

Periapical periodontitis, with persistent inflammation and failed bone healing after repeated routine root canal treatment, develops into refractory periapical periodontitis (RAP), which is a challenging condition to treat. RAP can result from the persistence of bacteria (1). Enterococcus faecalis is regarded as the predominant pathogen causing RAP, with prevalence values reaching up to 77% in persistent endodontic infections (2, 3). E. faecalis possesses various virulence factors, containing lipoteichoic acid (LTA), peptidoglycan, aggregation substance, cytolysin, and lytic enzymes. It can invade dentinal tubules, form biofilms, tolerate oligotrophic environments, and possess strong resistance to mechanical and chemical disinfection (4). There is a need for improved understanding of the pathogenesis of E. faecalis-induced RAP in order to devise novel treatment strategies.

Persistent inflammation is an important contributor to the delayed healing of RAP. Mild or moderate inflammation can trigger host defenses against invading pathogens and can maintain tissue homeostasis, whereas excessive inflammation may cause serious tissue damage and promote the progression of RAP. As the first line of defense of the immune system against pathogen infection, macrophages play a major role in regulating inflammatory response (5). It was found that both E. faecalis LTA and heat-killed antigens of E. faecalis were able to trigger the release of tumor necrosis factor-alpha (TNF-α) and nitric oxide in macrophages, aggravating the inflammatory response (6, 7). LTA could also activate the NLRP3 inflammasome in an NF-κB-dependent manner in macrophages (8). However, further understanding of how the modulatory effect of E. faecalis on macrophages affects the progression of RAP is needed.

Cell death is a common feature of the host immune response to bacterial infections. In contrast to apoptosis, a well-known form of nonlytic and immunologically silent regulated cell death, lytic cell death usually results in an inflammatory cytokine storm (9). Necroptosis is a recently identified form of lytic cell death which is highly proinflammatory and immunogenic (9, 10). Accumulating evidence has indicated that it is a tightly regulated pathway involving phosphorylation of receptor-interacting protein kinase 3 (RIPK3), which further triggers phosphorylation of mixed lineage kinase domain-like protein (MLKL), the necroptosis executioner. Phosphorylated MLKL (p-MLKL) oligomerizes and subsequently translocates to the plasma membrane, triggering membrane permeabilization, which is followed by the release of intracellular immunogenic contents that elicit inflammatory responses (11, 12).

Given its proinflammatory and immunomodulatory nature, the role of necroptosis in the pathogenesis of bacterial diseases has received increasing attention in recent years (13). Diverse bacteria could induce necroptosis of macrophages, promoting the progression of bacterial pneumonia (14, 15). Among oral bacterial infectious diseases, necroptosis was activated by Porphyromonas gingivalis during periodontitis development, and inhibition of necroptosis in such cases could attenuate inflammation as well as alveolar bone loss (16). Recently, we reported that E. faecalis could induce necroptosis of osteoblastic cells and that reduction in the osteoblast number might inhibit the repair efficiency of periapical tissues (17). The protein expression of p-MLKL was also reported to be upregulated in E. faecalis-infected macrophages (18). However, the role of necroptosis in RAP pathogenesis is not fully elucidated, and the extent to which E. faecalis-induced periapical lesions can be alleviated by inhibiting necroptosis remains unclear.

In this study, *in vivo* and *in vitro* experiments were conducted to identify the role of necroptosis in the progression of RAP. Periapical lesion specimens were collected to determine the occurrence of necroptosis in the human RAP lesions. *RIPK3*-deficient mice were then employed, and an experimental RAP model involving E. faecalis infection was established, to determine the involvement of necroptosis in the inflammatory response and bone destruction in RAP. Moreover, as we found that macrophages are crucial to necroptosis in RAP, an *in vitro* E. faecalis-infected model, as well as RIPK3 or MLKL inhibitor, was used to clarify the role of necroptosis in macrophages. This study provides novel insights into the pathogenesis of RAP and suggests potential therapeutic targets.

## RESULTS

### The RIPK3/MLKL signaling pathway is activated in periapical lesion specimens with RAP.

**FIG 1:**
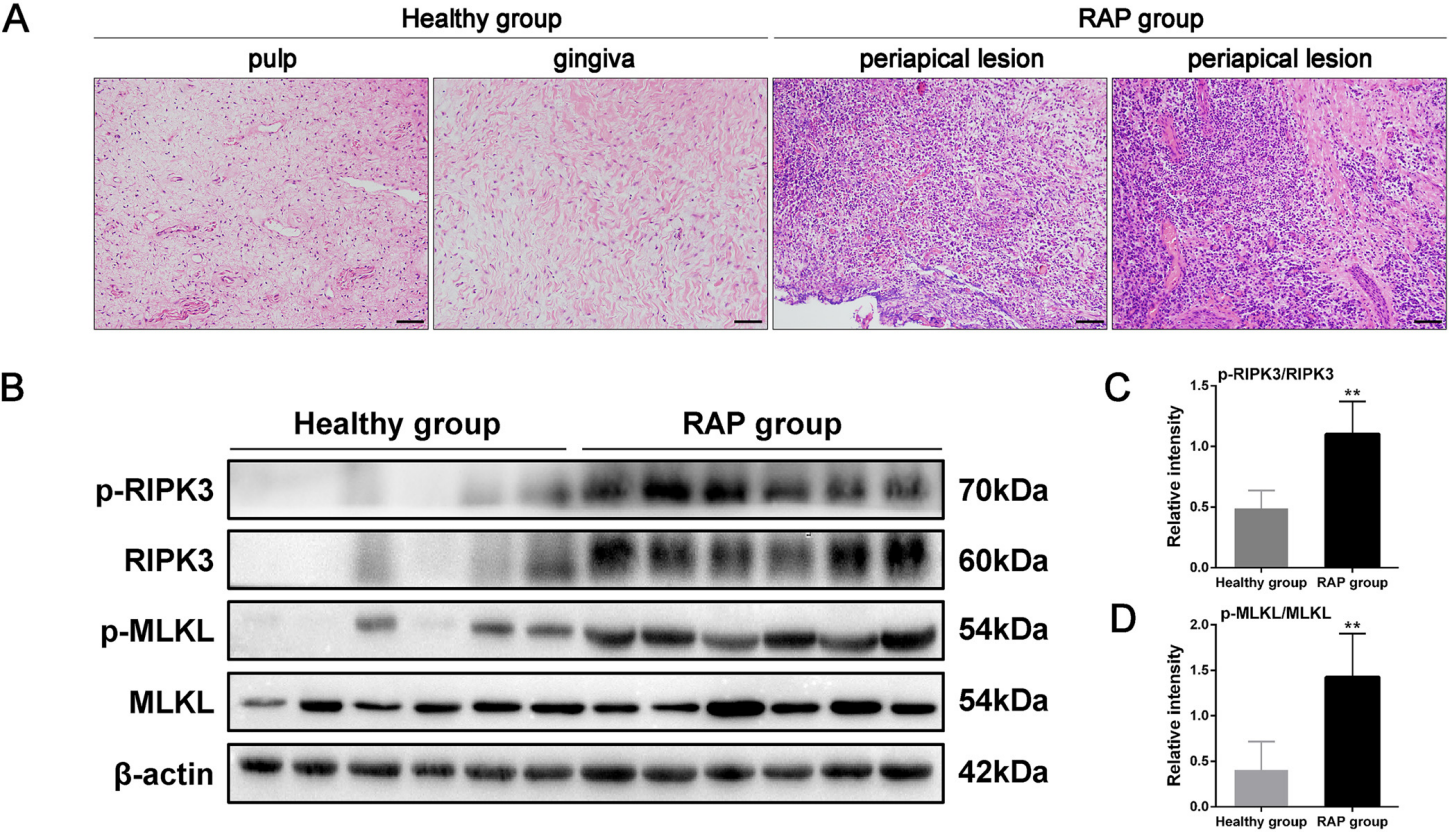
The RIPK3/MLKL signaling pathway is upregulated in periapical lesion specimens with refractory apical periodontitis (RAP). (A) Representative hematoxylin-eosin (HE) staining of healthy and RAP specimens from patients (*n* = 6/group) (200× magnification). Scale bar: 50 μm. (B) Immunoblot analysis of p-RIPK3, RIKP3, p-MLKL, and MLKL expression levels and (C and D) respective densitometric analysis in the specimens described above. β-Actin was the internal standard for protein loading (*n* = 6/group). Results are shown as mean ± standard deviation (SD) from three independent experiments. Statistical significance was determined using Student’s *t* test. *, *P < *0.05; **, *P* < 0.01; ***, *P* < 0.001.

To evaluate whether necroptosis is implicated in the pathogenesis of RAP, periapical lesion specimens were collected from patients diagnosed with RAP. Hematoxylin and eosin (HE) staining showed that the RAP group had more cellular infiltration than the control group (Fig. 1A). The expression and distribution of p-MLKL, a necroptosis-related marker, were then determined by immunohistochemistry. RAP specimens stained positive for p-MLKL, while p-MLKL was barely detectable in healthy tissues (see Fig. S1A in the supplemental material). In addition, the specimens that displayed upregulation of p-MLKL also stained positive for interleukin 1β (IL-1β), IL-6, and tumor necrosis factor α (TNF-α) (Fig. S1B). Immunoblot analysis further revealed that the p-RIPK3/RIPK3 and p-MLKL/MLKL expression ratios were significantly elevated in RAP periapical lesion specimens (*P < *0.01) compared to those in the healthy specimens, suggesting activation of the RIPK3/MLKL signaling pathway in RAP (Fig. 1B to D).

Collectively, these findings reveal a positive association between necroptosis and inflammation, indicating that necroptosis may play a role in the progression of RAP.

### *RIPK3* knockout inhibits E. faecalis-induced necroptosis in an experimental RAP mouse model.

**FIG 2:**
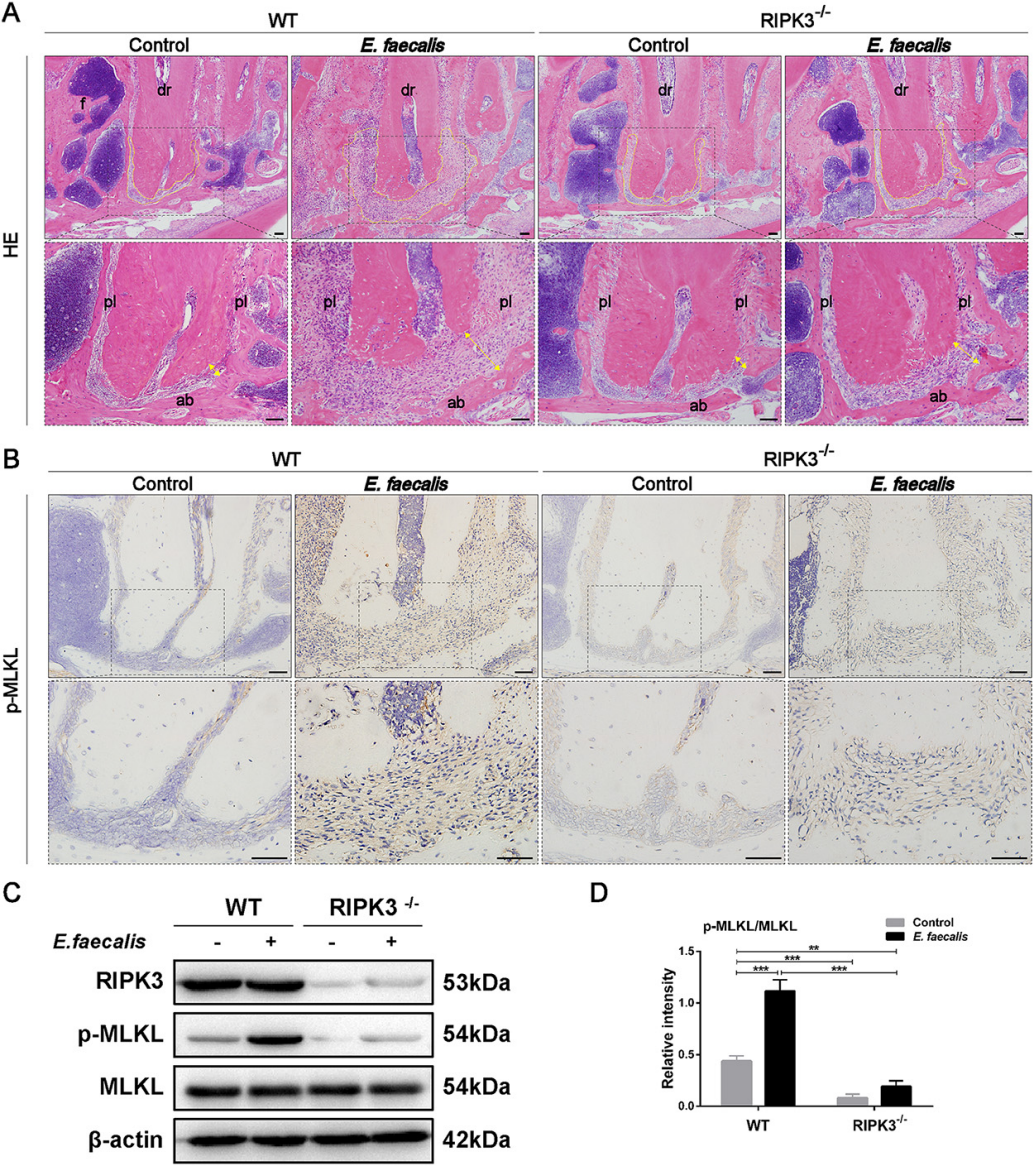
Knockout of *RIPK3* suppresses Enterococcus faecalis-induced necroptosis in an experimental refractory apical periodontitis (RAP) mouse model. (A) Representative hematoxylin-eosin staining of the apical region in wild-type (WT) and *RIPK3*^−/−^ mice with or without E. faecalis infection (*n* = 6/group; 100× and 200× magnification). The yellow double arrows mark width of the periodontal membrane; yellow dotted lines mark the periapical area; dr, distal root of the mandibular first molar; ab, alveolar bone; pl, periodontal ligament; f, furcation area. (B) Representative immunohistochemical staining of p-MLKL in the apical region of mandibular first molars (*n* = 6/group; 200× and 400× magnification). Scale bar: 50 μm. (C) Immunoblot analysis of RIKP3, p-MLKL, and MLKL expression levels and (D) densitometric analysis. Results are shown as mean ± SD from three independent experiments. Statistical significance was determined using one-way analysis of variance (ANOVA) followed by the least significant difference (LSD) *post hoc* test. *, *P* < 0.05; **, *P* < 0.01; ***, *P < *0.001.

With the purpose of exploring the role of necroptosis in RAP progression *in vivo*, *RIPK3*-deficient mice were employed. An experimental RAP mouse model was established by injecting E. faecalis (ATCC 29212 strain), the predominant causative organism of RAP, into the mandibular first molars of *RIPK3*^−/−^ mice and control wild-type (WT) mice. Histopathological changes in periapical lesions were evaluated using HE staining. The unexposed first molars of WT and *RIPK3*^−/−^ mice displayed similarly preserved periapical structures. Intriguingly, periapical lesion formation was observed after model induction in WT mice, whereas inflammatory cell infiltration and disruption of periapical tissues were markedly attenuated in *RIPK3*^−/−^ mice (Fig. 2A). Consistently, the executioner of necroptosis, p-MLKL, was strongly expressed in the periapical lesions of E. faecalis-infected teeth in WT mice, whereas it was weakly expressed in RIPK3^−/−^ mice, as demonstrated by immunohistochemistry (Fig. 2B). In addition, immunoblot analysis also indicated that the phosphorylation of MLKL was markedly enhanced in WT mice after model induction but was significantly inhibited in *RIPK3^−/−^* mice (Fig. 2C and D).

### *RIPK3* knockout attenuates inflammatory bone loss in an E. faecalis-infected RAP mouse model.

**FIG 3:**
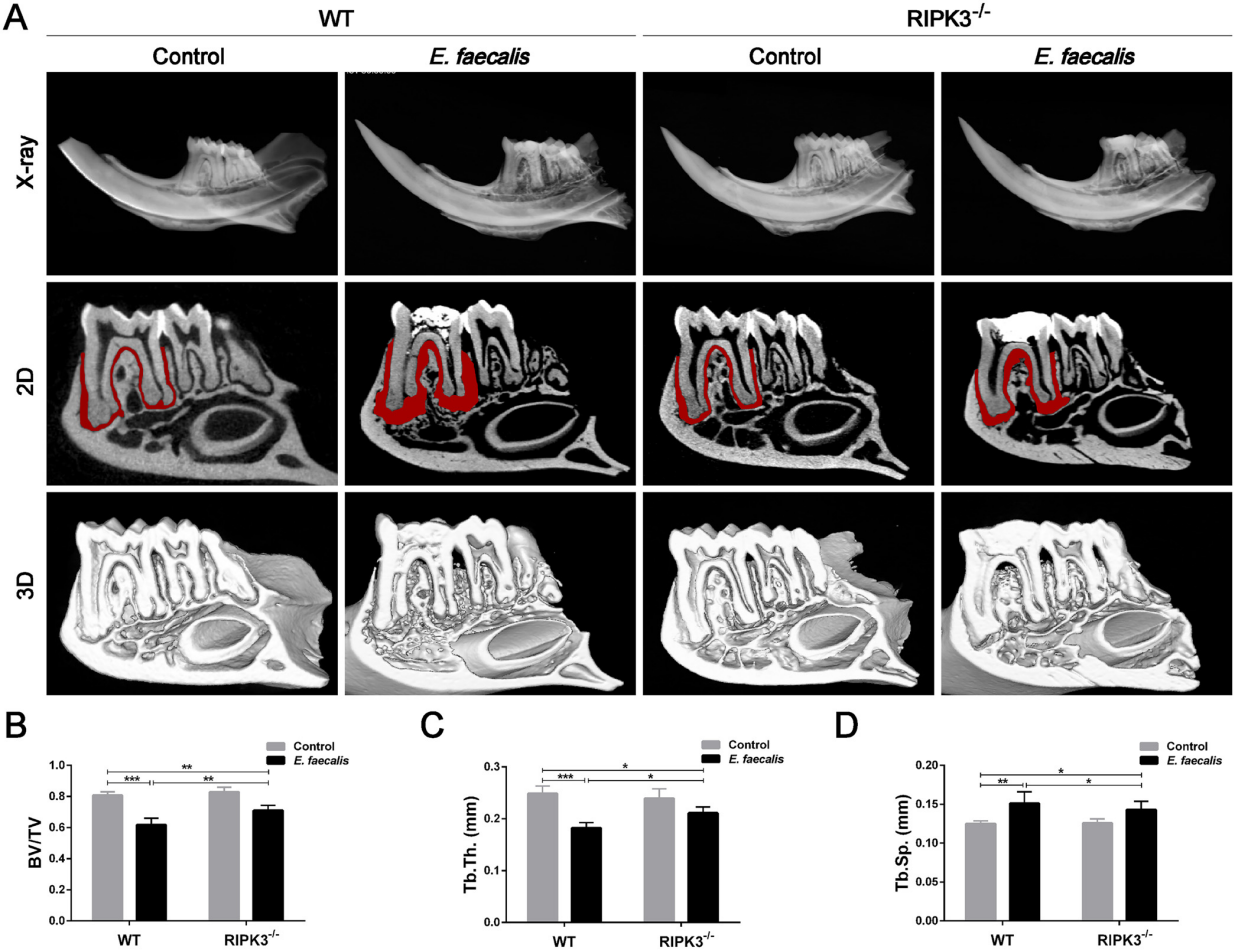
Knockout of *RIPK3* alleviates bone destruction in an Enterococcus faecalis-infected refractory apical periodontitis (RAP) mouse model. (A) Micro-CT evaluation of bone destruction in the apical region of mandibular first molars in wild-type (WT) and *RIPK3*^−/−^ mice with or without E. faecalis infection. Representative X-ray images and 2D images as well as 3D reconstruction. The red areas mark bone destruction. (B) Evaluation of the bone volume fraction of the residual alveolar bone. (C and D) Microstructural parameter analysis of the trabecular bone, including (C) trabecular thickness (Tb. Th) and (D) trabecular bone clearance (Tb. sp) (*n* = 6/group). Results are shown as mean ± SD from three independent experiments. Statistical significance was determined using one-way ANOVA with the LSD *post hoc* test. *, *P < *0.05; **, *P < *0.01; ***, *P < *0.001.

To substantiate the impact of necroptosis in E. faecalis-infected RAP mice, periapical inflammation and bone destruction were assessed. Immunohistochemistry revealed that expression levels of IL-1β, IL-6, and TNF-α were greatly elevated in the periapical area of E. faecalis-infected molars in WT mice compared to those in the control. In contrast, periapical lesions in *RIPK3*^−/−^ mice showed decreased expression of inflammatory cytokines (Fig. S2). Microcomputed tomography (micro-CT) analysis revealed that *RIPK3* deletion *per se* (without E. faecalis infection) had no significant effect on bone destruction, and the bone parameters of the uninfected WT and *RIPK3^−/−^* mice were similar. However, bone destruction in the periapical area of the mandibular first molar was markedly alleviated by RIPK3 deficiency during E. faecalis infection (Fig. 3A, Fig. S3). Moreover, the apical bone area of the infected first molar in *RIPK3*^−/−^ mice demonstrated significantly increased bone density, characterized by an increased bone volume/tissue volume (BV/TV) ratio, increased trabecular thickness (Tb. Th), and decreased trabecular separation (Tb. Sp) compared to those in the WT mice (Fig. 3B to D).

Taken together, the *in vivo* results indicated that the activation of necroptosis in E. faecalis-infected RAP mouse model and inhibition of necroptosis by RIPK3 deficiency could alleviate inflammation and bone destruction.

### Necroptosis is activated in macrophages in human and murine RAP lesions.

**FIG 4:**
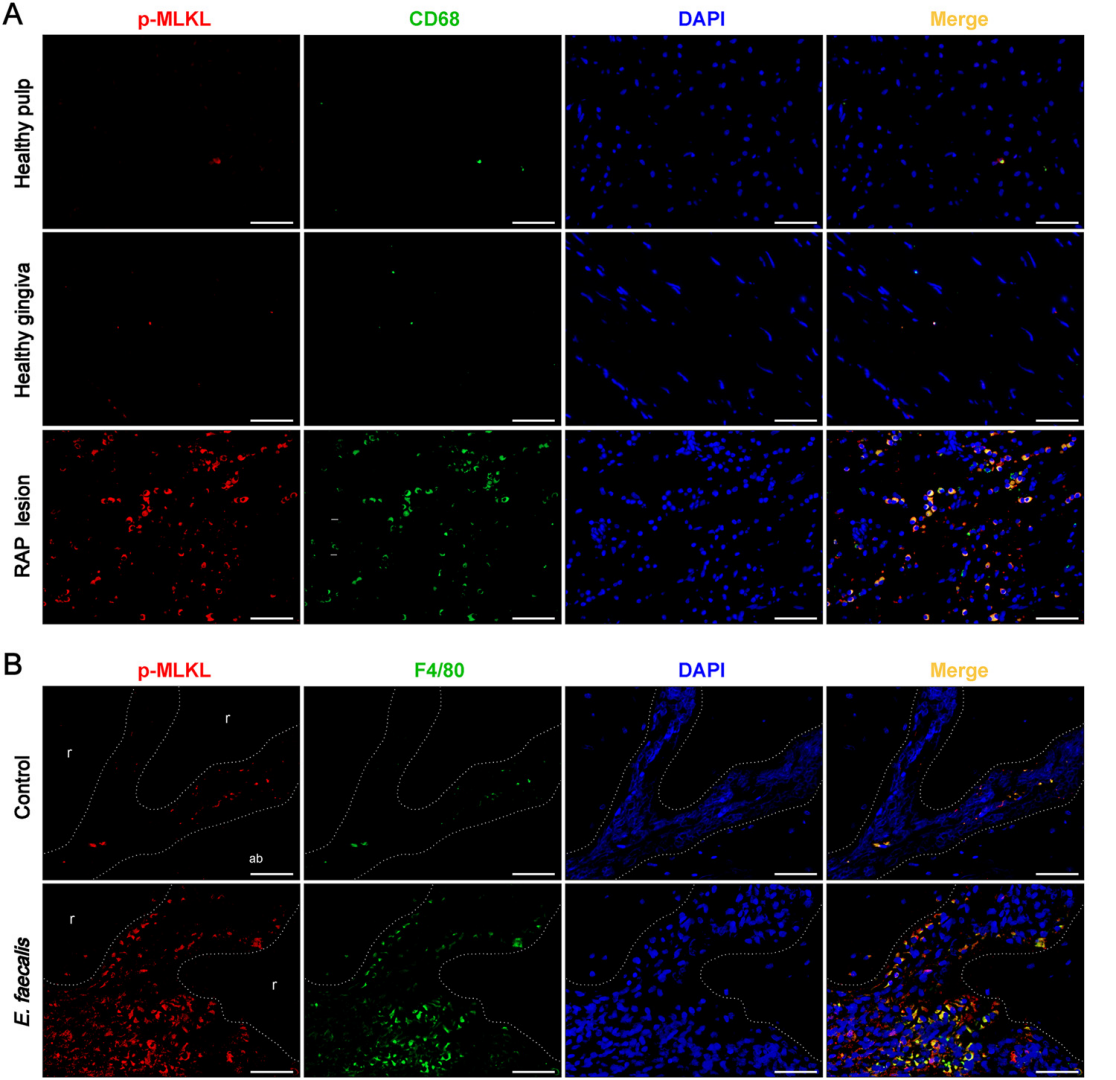
The executioner of necroptosis colocalizes with a macrophage marker in refractory apical periodontitis (RAP) lesions. (A) Double-immunofluorescence staining of p-MLKL (red), executioner of necroptosis, and the macrophage marker CD68 (green) in healthy and RAP specimens from patients (*n* = 6/group). (B) Immunofluorescence colocalization of p-MLKL (red) and macrophage marker F4/80 (green) in uninfected and Enterococcus faecalis-infected RAP mouse models (*n* = 6/group). DAPI staining shows cell nuclei (blue; 400× magnification). Scale bar: 50 μm. The representative images were obtained from three independent experiments.

Considering that macrophages are the first line of defense of the immune system, we performed double-labeling immunofluorescence in human and murine RAP lesions to explore whether macrophages are an important cell type in necroptosis contributing to RAP. The number of macrophages infiltrating the apical tissues in RAP lesions was appreciably higher than that in the normal gingiva and pulp. Notably, p-MLKL, the executioner of necroptosis, was predominantly expressed in CD68^+^ macrophages (Fig. 4A). Consistently, in periapical lesions of E. faecalis-infected RAP models in mice, colocalization of p-MLKL and F4/80, a marker of mature murine macrophages, was observed. Conversely, colocalization was seldom observed in the uninfected control group (Fig. 4B).

Collectively, these findings suggest that the activation of necroptosis in macrophages may be involved in the pathogenesis of RAP.

### Live E. faecalis infection induces RIPK3/MLKL-mediated necroptotic death in macrophages.

**FIG 5:**
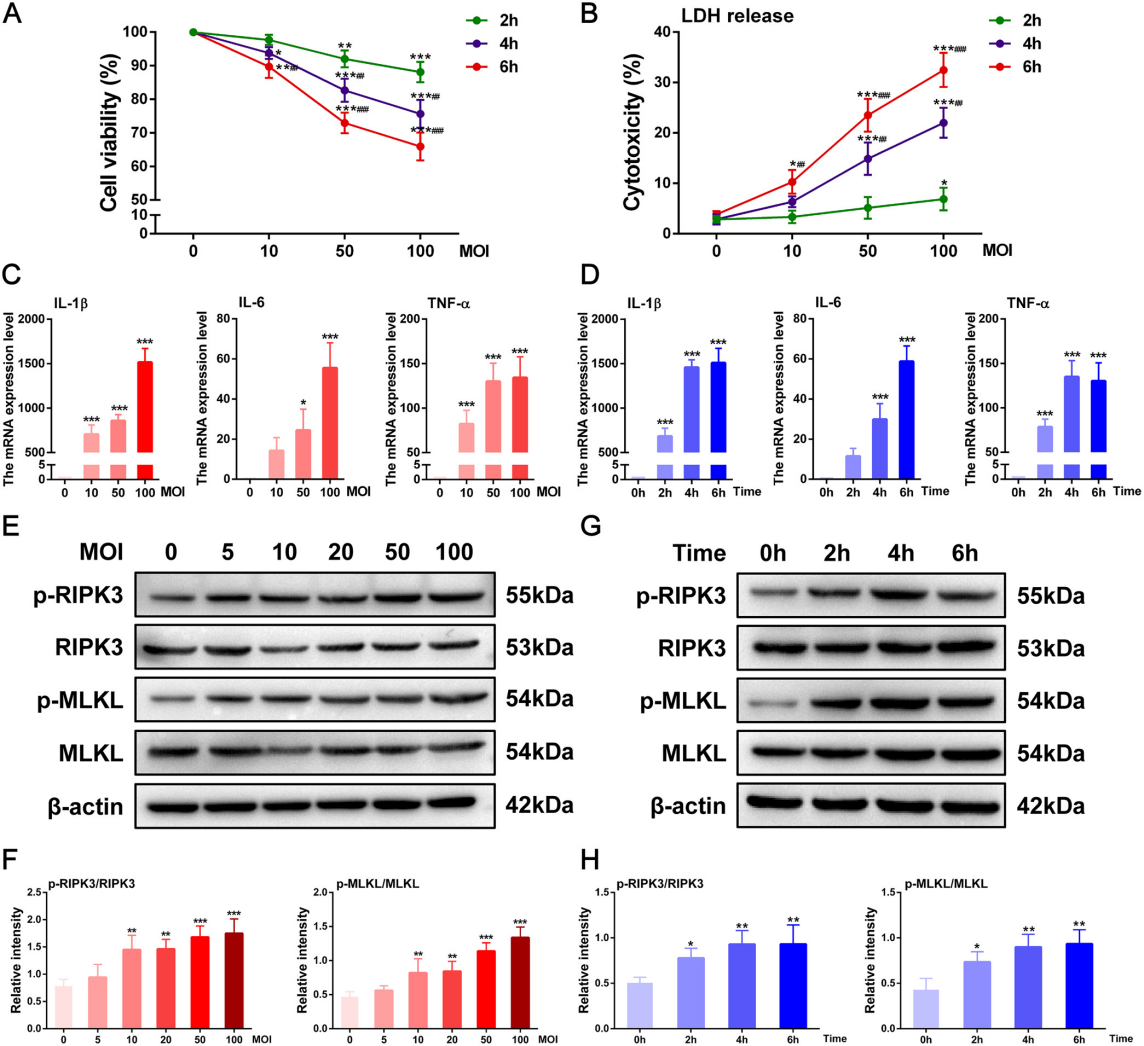
Enterococcus faecalis infection induces necroptotic cell death of macrophages. (A) Effect of E. faecalis on the viability of RAW264.7 cells. (B) Lactate dehydrogenase release by infected cells. (C and D) Expression of mRNA of inflammatory cytokines, including IL-1β, IL-6, and TNF-α, at different (C) multiplicities of infection (MOIs) and (D) times. (E) Immunoblot analysis of p-RIPK3, total RIKP3, p-MLKL, and total MLKL in E. faecalis-infected cells with various MOI values for 6 h and (F) respective densitometric analyses. (G) Immunoblot analysis of the above-described necroptosis markers in RAW264.7 cells infected with E. faecalis at an MOI of 100 at different time intervals and (H) respective densitometric analyses. β-actin was the internal standard for protein loading. Results are shown as mean ± SD from three replicates from three independent experiments. Statistical significance was determined using one-way ANOVA with LSD *post hoc* test. *, *P < *0.05; **, *P < *0.01; ***, *P < *0.001. #, *P* < 0.05; ##, *P* < 0.01; ###, *P* < 0.001 versus the infection group with the same MOI at 2 h (A and B).

To determine whether necroptosis is activated in macrophages during E. faecalis infection, RAW264.7 cells were infected with E. faecalis to establish an *in vitro* infection model. E. faecalis markedly inhibited cell viability and elevated lactate dehydrogenase (LDH) release in a multiplicity of infection (MOI)- and time-dependent manner (Fig. 5A and B). Additionally, the expression of the inflammatory markers IL-1β, IL-6, and TNF-α was significantly upregulated by E. faecalis infection (Fig. 5C and D). The phosphorylation levels of the necroptosis-associated proteins RIPK3 and MLKL also tended to increase with increasing MOI and exposure time (Fig. 5E to H).

**FIG 6:**
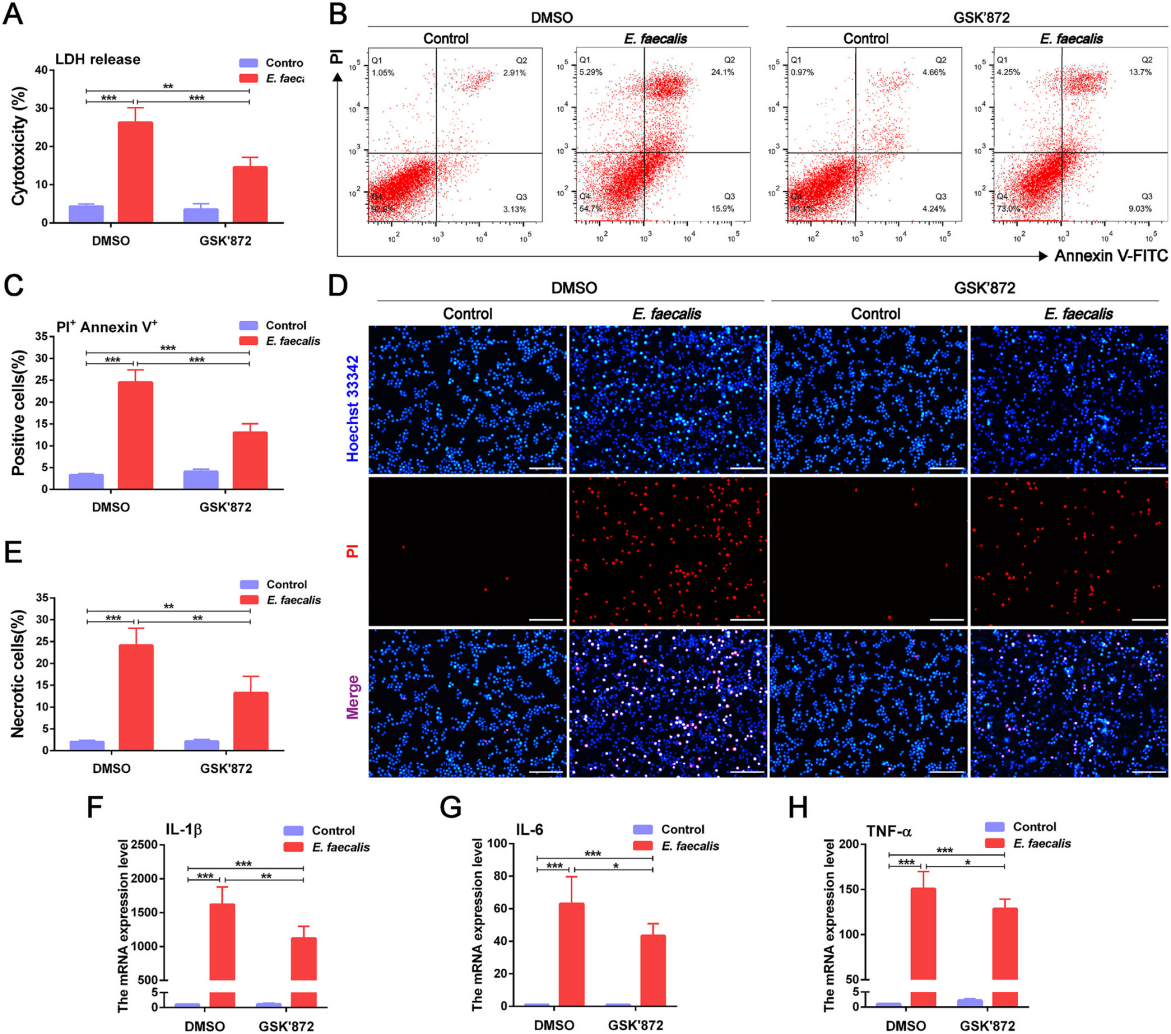
Pretreatment with RIPK3 inhibitor reduces cell death and inflammation during Enterococcus faecalis infection. (A) Lactate dehydrogenase release analysis of E. faecalis-infected RAW264.7 cells with an MOI of 100 for 6 h in the presence or absence of GSK’872, the RIPK3 inhibitor. (B) Flow cytometric analysis of propidium iodide (PI) and annexin V staining of cells and (C) the quantification of cells double-positive for PI and annexin V staining. (D) Representative fluorescence images of Hoechst 33342 (blue) and PI (red) double staining and (E) quantitative analysis of necrotic cells (200× magnification). Scale bar: 100 μm. (F to H) The mRNA expression levels of inflammatory cytokines, including (F) IL-1β, (G) IL-6, and (H) TNF-α. Results are shown as mean ± SD from three replicates from three independent experiments. Statistical significance was determined using one-way ANOVA with LSD *post hoc* test. *, *P < *0.05; **, *P < *0.01; ***, *P < *0.001.

To further identify the contribution of necroptosis to E. faecalis-induced cell death and inflammation, RIPK3 inhibitor GSK’872 or MLKL inhibitor GW806742X was administered prior to E. faecalis infection. It was found that pretreatment with GSK’872 or GW806742X significantly decreased LDH release in E. faecalis-infected cells at 100 MOI for 6 h (*P < *0.001) (Fig. 6A, Fig. S4A). In addition, the population of infected cells double-positive for annexin V and propidium iodide (PI) staining also showed a significant reduction in the presence of GSK’872 or GW806742X (*P < *0.001) (Fig. 6B and C, Fig. S4B and C). Similarly, Hoechst 33342/PI double staining indicated that RIPK3 or MLKL inhibition significantly reduced the proportion of necrotic cells in response to E. faecalis infection (*P < *0.01) (Fig. 6D and E, Fig. S4D and E). In terms of the effect of RIPK3 or MLKL inhibition on macrophage inflammation, pretreatment with the RIPK3 or MLKL inhibitor markedly decreased the expression of inflammatory cytokine genes triggered by E. faecalis infection (Fig. 6F to H, Fig. S4F to H).

Taken together, these results demonstrate that E. faecalis triggers necroptosis of macrophages through the RIPK3/MLKL signaling pathway, which is implicated in inflammatory responses.

## DISCUSSION

The interplay between pathogenic microorganisms and the immune response leads to the activation of cell death, which affects progression of the diseases. Necroptosis, a highly proinflammatory form of regulated cell death, is found to be implicated in the pathogenesis of various infectious diseases. We investigated the role of necroptosis in RAP progression and revealed that necroptosis is activated in human RAP specimens and in the experimental RAP model in mice and that it mainly involves macrophages. Inhibition of necroptosis by *RIPK3*-knockout in mice and by GSK’872 or GW806742X administration in RAW264.7 macrophages markedly alleviated the inflammatory response.

The role of necroptosis during bacterial infection has been increasingly appreciated in recent years. The present study revealed that E. faecalis-induced necroptosis promotes the development of RAP, based on an analysis of human clinical specimens, animal models, and cellular experiments. Consistently, necroptosis was also reported to contribute to the formation of cardiac microlesions during severe bacteremic E. faecalis infection in mice (19). With the release of death-associated molecular patterns, necroptotic cells can transmit danger signals and activate inflammatory responses. Macrophages are important mediators of host inflammatory responses to infection (20). In the current study, we found abundant macrophage infiltration in periapical lesion specimens from RAP-diagnosed patients as well as from an experimental RAP model. There was strong colocalization between the necroptosis-related markers, p-MLKL, and the markers of macrophages, CD68 and F4/80. In agreement with our results, several recently published studies also demonstrated that macrophage necroptosis was involved as part of the mechanism of enhancing inflammatory responses (21–23).

The core components of the necroptotic pathway are RIPK3 and MLKL, while RIPK1 requirement can be bypassed (24). Necroptosis is generally evaluated by detecting the phosphorylation states of RIPK3 and MLKL (25). In particular, the phosphorylation of MLKL, a downstream factor of RIPK3, is considered to be a specific marker for necroptosis. Our study indicated that the expression levels of p-MLKL were remarkably elevated in clinical specimens from patients diagnosed with RAP, E. faecalis-infected RAP mouse model, and E. faecalis-infected macrophages, suggesting the involvement of necroptosis in RAP progression. Similarly, protein expression of p-MIKL was also found to be upregulated in macrophages during E. faecalis (OG1RF strain and two root canal-isolated strains) infection (18). Additionally, histopathology of the collected periapical lesion specimens in this study, positively stained for p-MLKL, mainly showed periapical granulomas and abscesses. Exploring the role of necroptosis in RAP of different pathological types may be an interesting direction for future research.

Notably, recent studies have suggested that the phosphorylation of MLKL by itself may be insufficient to prove the activation of necroptosis (24), as it failed to induce host cell death during *Listeria* infection (26). Hence, it is necessary to use inhibitor or knockdown approaches to determine the involvement of necroptosis. Therefore, we utilized *RIPK3*-deficient mice *in vivo* and GSK’872, a specific RIPK3 inhibitor, as well as GW806742X, a specific MLKL inhibitor, *in vitro*. The use of *RIPK3*-deficient mice, GSK’872, and GW806742X in our study supported the notion that macrophage necroptosis is activated during E. faecalis infection. Bone destruction is an essential feature in the diagnosis and prognosis of apical periodontitis (27). We observed an apparent reduction in periapical bone loss induced by E. faecalis in *RIPK3*-deficient mice. Consistently, bone loss was also alleviated in Fusobacterium nucleatum-induced experimental apical periodontitis in mice after inhibition of RIPK3 using an adeno-associated virus (28). Interestingly, in addition to its well-established roles in necroptosis, RIPK3 can also serve as a regulator of inflammation and cell death, independent of necroptotic function. Recent studies have shown that RIPK3 promotes the activation of NLRP3 inflammasomes and contributes to inflammation in an MLKL-independent manner (29, 30). The combined application of *MLKL*-deficient mice in future studies is recommended to provide more robust evidence of the involvement of necroptosis in RAP progression. Nevertheless, the current study identified a critical role of RIPK3 in E. faecalis-induced inflammation and tissue injury, suggesting that RIPK3 may be a promising target for RAP therapy.

Necroptosis is regarded as a form of non-caspase-dependent regulated cell death, which occurs in the absence of caspase-8 activation (31). Some pathogens have developed strategies to inhibit caspase-8 activity, resulting in the activation of necroptosis (9, 32). In this study, E. faecalis induced macrophage necroptosis without the requirement for exogenous caspase inhibitors. It is possible that live E. faecalis, which is highly immunogenic, may express caspase-8 inhibitors that directly precipitate necroptosis. Additionally, caspase-8 has been found to be the molecular switch that controls pyroptosis, apoptosis, and necroptosis (33). PANoptosis, a newly emerging concept highlighting the cross talk and coordination between pyroptosis, apoptosis, and necroptosis, has been proposed (34). Recently, we have reviewed the functional consequences and activation mechanisms of PANoptosis in oral infectious diseases (20). E. faecalis infection can induce pyroptosis, apoptosis, and necroptosis of macrophages based on previous and current studies (18, 35, 36). However, in addition to the detection of simultaneous activation of the three regulated cell death pathways, the identification of a PANoptosome, a single molecular complex composed of molecules from the pyroptotic, apoptotic, and necroptotic cell death pathways, has been recommended to determine PANoptosis (37). Whether E. faecalis infection could promote the formation of the PANoptosome is unclear, and the complete composition and mechanisms governing the assembly of the PANoptosome remain unknown. Our study hints that RIPK3 might be involved in this process.

**FIG 7:**
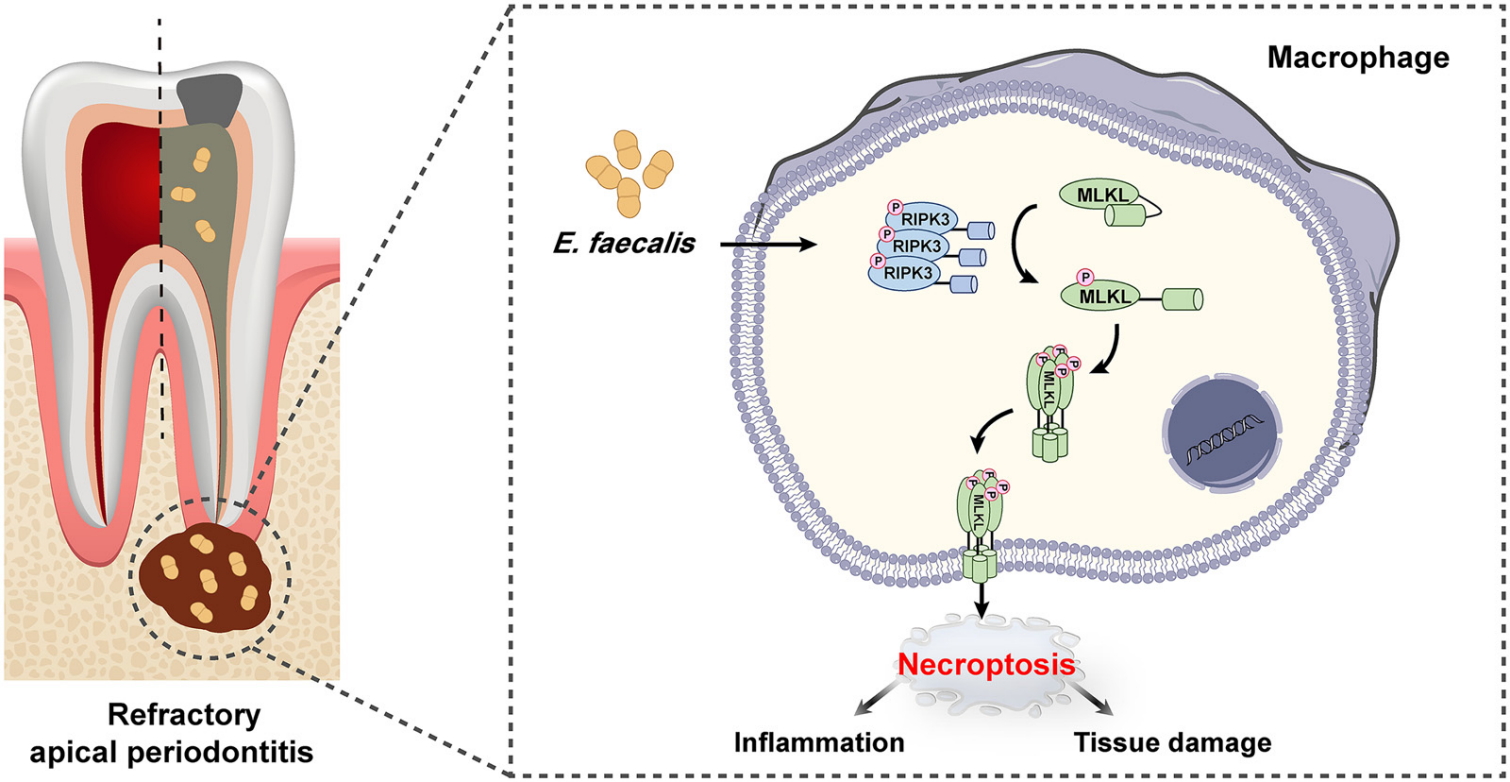
Schematic model for the Enterococcus faecalis-induced macrophage necroptosis promoting refractory apical periodontitis. E. faecalis infection promotes the phosphorylation of RIPK3 in macrophages, which further recruits and phosphorylates MLKL, the executioner of necroptosis. Phosphorylated MLKL oligomerizes and translocates to the cell membrane, leading to membrane rupture and release of intracellular immunogenic contents. The activation of macrophage necroptosis subsequently triggers the inflammatory response and tissue destruction, promoting refractory apical periodontitis (RAP) progression.

In conclusion, we identified an important role for RIPK3/MLKL-mediated macrophage necroptosis induced by E. faecalis in the pathogenesis of RAP (Fig. 7). These findings suggest that manipulating macrophage necroptosis represents a potential therapeutic approach for preventing E. faecalis-induced periapical lesions. The detailed mechanism by which macrophage necroptosis promotes inflammation during this process warrants further clarification. Additionally, another function of macrophages is eliminating invading pathogens. The impact of macrophage necroptosis on the clearance of E. faecalis in the pathogenesis of RAP also deserves further study.

## MATERIALS AND METHODS

### Sample collection.

Twelve periapical lesion specimens were obtained from the teeth of patients diagnosed with RAP during endodontic microsurgery. As the controls, six healthy dental pulp and gingival samples were derived from caries-free permanent teeth extracted for orthodontic reasons, according to a previous study (38). Patients were excluded if they had systemic diseases, had undergone antibiotic therapy, or were currently pregnant or lactating. Half of the samples were immediately fixed for histology, while the others were snap-frozen and stored at −80°C for protein extraction. All samples were obtained after obtaining informed consent from participants, and the study was approved by the Ethics Committee of Nanfang Hospital, Southern Medical University.

### Bacterial culture.

E. faecalis (ATCC 29212 strain) was employed as described previously (17). In brief, the bacteria were cultured in brain-heart infusion broth (BD Difco) at 37°C overnight. The concentration of bacteria was standardized to an optical density (OD) of 0.5 at 600 nm, corresponding to 2 × 10^8^ CFU/mL.

### Animals and induction of RAP.

Wild-type C57BL/6 mice were obtained from the Experimental Animal Center of Southern Medical University. RIPK3-deficient (*RIPK3*^−/−^) mice on a C57BL/6 background were obtained from Genentech (39). All animal experiments were approved by the Ethics Committee of Nanfang Hospital, Southern Medical University. To model RAP, 8-week-old mice were anesthetized with pentobarbital sodium (50 mg/kg) via intraperitoneal injection. The left mandibular first molars were maintained as an internal control, while the right mandibular first molars were exposed with a dental handpiece and 1/4 ball drill and were filled with E. faecalis (1.5 × 10^9^ CFU/mL) using microliter syringes (Gaoge). Afterward, the access cavity was sealed with glass ionomer cement (Fuji IX; GC Corporation). The mice were fed regularly and were sacrificed 28 days after surgery. Mandibles were isolated, hemisected, and further fixed for microcomputed tomography and histology or immediately frozen and stored at −80°C for protein extraction.

### Micro-CT analysis.

The fixed mandibles were scanned with a microcomputed tomography system (μCT 80; Scanco Medical AG). The region of interest was restricted to the alveolar bone surrounding the mandibular first molars with reference to a previous study (40). For visualization, three-dimensional (3D) digitized images were created using 3D reconstruction software (Avatar; PINGSENG Healthcare). The 3D analysis was also performed in the selected region of interest to determine the BV/TV, Tb.Th, and Tb.Sp. Each group contained three individual samples.

### Histological and immunohistochemistry staining.

The clinical specimens were processed for paraffin embedding, whereas the murine mandibles were embedded in paraffin after decalcification with 10% EDTA. Briefly, all samples were subjected to routine histological processing, including dehydration in increasing concentrations of alcohol, diaphanization in xylol, embedding in paraffin, and sectioning at 4-μm thickness. Slices were subjected to HE staining for histopathological evaluation after deparaffinization and rehydration. For immunohistochemical analysis, prepared slices were pretreated with H_2_O_2_ and blocked with goat serum after dewaxing, rehydration, and antigen retrieval. Next, the slices of clinical specimens were incubated with primary antibodies against p-MLKL (Abcam; Invitrogen), IL-1β (Abclonal), IL-6 (Abclonal), and TNF-α (Abclonal). They were then probed with the corresponding horseradish peroxidase-conjugated secondary antibodies (Jackson ImmunoResearch; ZSGB-BIO). After DAB (3,3'-diaminobenzidine tetrahydrochloride) staining, an optical microscope (Olympus) was used for optical imaging.

### Immunofluorescence staining.

The sections of clinical specimens were incubated with the primary antibody against p-MLKL (Abcam) and CD68 (Abcam), while murine sections were incubated with antibodies for p-MLKL (Invitrogen) and F4/80 (Abcam) at 4°C overnight. After rinsing with phosphate-buffered saline (PBS), the sections were labeled with fluorescently labeled secondary antibodies, including Alexa Fluor 594 goat anti-rabbit (ZSGB-BIO), Alexa Fluor 488 goat anti-mouse (ZSGB-BIO), and Alexa Fluor 488 goat anti-rat (Abcam). Nuclei were counterstained with DAPI (4′,6-diamidino-2-phenylindole; Sigma-Aldrich). Fluorescence images were captured using a fluorescence microscope (Zeiss).

### Cell culture and treatment.

RAW264.7 cells, which are murine macrophages, were obtained from ATCC and were cultured in Dulbecco’s modified Eagle’s medium (Gibco) supplemented with 10% fetal bovine serum (FBS; Gibco) in a suitable incubator. To determine the impact of E. faecalis infection, RAW264.7 cells were treated with live E. faecalis at the desired MOI for the indicated time. GSK’872, a specific inhibitor of RIPK3, and GW806742X, a specific MLKL inhibitor, were used to further identify the involvement of necroptosis. Specifically, the cells were pretreated with 10 μM GSK’872 (Selleckchem) or 2 μM GW806742X (MedChemExpress) 2 h prior to E. faecalis infection, while an equal volume of dimethyl sulfoxide was applied as the control.

### Cell viability and cytotoxicity assay.

RAW264.7 cells were seeded in 96-well culture plates at a density of 6 × 10^4^ cells/well and then treated with E. faecalis at an MOI of 0, 10, 50, and 100 for 2, 4, and 6 h. Wells with untreated RAW264.7 cells served as the control, while wells containing only E. faecalis with the corresponding MOI served as the blank infection group. A CCK8 cytotoxicity assay kit (Dojindo Laboratories) was applied to perform the cell viability assay, while the cytotoxicity was evaluated with a lactate dehydrogenase (LDH) assay kit (Beyotime), following the manufacturer’s protocol. The corresponding cell viability and cytotoxicity were calculated as described previously (17).

### Western blotting.

Protein lysates were obtained using radioimmunoprecipitation assay (RIPA) buffer supplemented with the protease and phosphatase inhibitor cocktail (Bimake). Grinding and ultrasonication were additionally used for protein extraction from the clinical specimens and murine mandibles. Protein was quantified using the bicinchoninic acid (BCA) protein assay kit (Thermo Fisher Scientific). Extracts of 20 μg of total protein were subjected to 10% sodium dodecyl sulfate-polyacrylamide gel electrophoresis and were transferred onto the polyvinylidene fluoride membranes (Bio-Rad). After being blocked, the membranes were incubated with specific primary antibodies for RIPK3 (1:500, Santa Cruz Biotechnology; 1:1,000, Novus Biologicals), p-RIPK3 (1:1,000, Abcam; 1:1,000, Cell Signaling Technology), MLKL (1:1,000, Abcam; 1:1,000, Cell Signaling Technology), p-MLKL (1:1,000, Abcam; 1:1,000, Cell Signaling Technology), and β-actin (1:5,000, Abclonal) at 4°C overnight. The membranes were then incubated with the secondary antibodies conjugated with horseradish peroxidase (1:5,000, Proteintech). Afterward, the membranes were visualized using bio-Femto enhanced chemiluminescence (Fudebio) and a chemiluminescent imaging system (Tanon 5200). ImageJ software was used for the analysis of optical density of the immunoblot bands.

### Reverse transcription-quantitative PCR.

Total RNA was extracted from RAW264.7 cells with RNAiso Plus (TaKaRa) and was quantified using a Nanodrop (Thermo Fisher Scientific). Subsequently, a reverse transcriptase kit (TaKaRa) was applied to synthesize cDNA through reverse transcription of RNA. Quantitative real-time PCR amplification was carried out with SYBR Premix *Ex Taq* (TaKaRa) using a LightCycler 480 system (Roche). The relative gene expression levels of IL-1β, IL-6, and TNF-α were normalized to the expression of β-actin and quantified using the 2^−ΔΔ^*^CT^* method. Primer sequences used in this study are available in Table S1 in the supplemental material.

### Flow cytometry analysis.

The RAW264.7 cells, seeded at a density of 2 × 10^6^ cells per well in 6-well cell culture plates, were treated with E. faecalis at an MOI of 100 for 6 h, with or without the presence of GSK’ 872 or GW806742X. Afterward, the cells were collected, rinsed with PBS, and then stained with propidium iodide (PI) and fluorescein isothiocyanate-conjugated annexin V (KeyGEN). The percentage of double-positive cells was analyzed with FACSCalibur flow cytometer (BD Biosciences) and FlowJo software.

### Hoechst 33342/PI fluorescent staining.

The treated cells were gently washed with PBS and then double stained with Hoechst 33342 and PI (Solarbio) at 4°C for 30 min, protected from light, according to the kit instruction. A fluorescence microscope (Olympus) was subsequently employed to capture the images. The necrotic cells were characterized by strong bright blue and red fluorescence, whereas normal cells displayed uniform dark blue fluorescence.

### Statistical analysis.

The experiments were carried out in at least three independent triplicates. Data are expressed as the mean ± standard deviation. Statistical analysis was performed using SPSS software (version 23.0; IBM SPSS Inc.). Two-group comparisons were conducted using Student’s *t* test. One-way analysis of variance (ANOVA), followed by a *post hoc* test with the least significant difference (LSD) was applied for multiple-group comparisons. Two-tailed *P* value of <0.05 was considered statistically significant.

### Data availability.

The data generated during the current study are available from the corresponding author on reasonable request.
