# Supplementary material for: Enterococcus faecalis-Induced Macrophage Necroptosis Promotes Refractory Apical Periodontitis
Source: Microbiol Spectr. 2022 Jun 16;10(4):e01045-22. doi: 10.1128/spectrum.01045-22 (PMC9431707; doi:10.1128/spectrum.01045-22)
Supplement: Supplemental file 1 — Fig. S1 to S4; Table S1. Download spectrum.01045-22-s0001.pdf, PDF file, 0.9 MB [file spectrum.01045-22-s0001.pdf]

Supplemental material

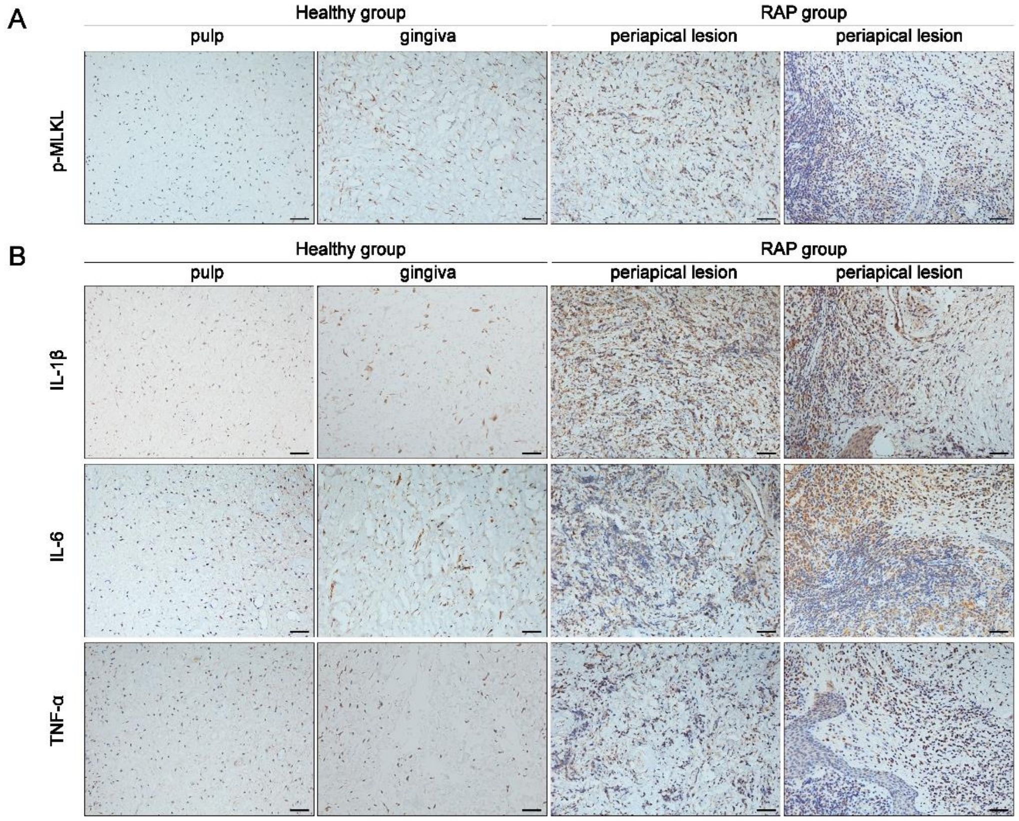

Figure S1 Elevated expression of p-MLKL and inflammatory cytokines in periapical lesion specimens with refractory apical periodontitis (RAP). Representative immunohistochemical staining of p-MLKL (A) and inflammatory cytokines, including IL-1β, IL-6, and TNF-α (B), in healthy and RAP specimens from patients (n = 6/group) (200× magnification). Scale bar: 50 μm.

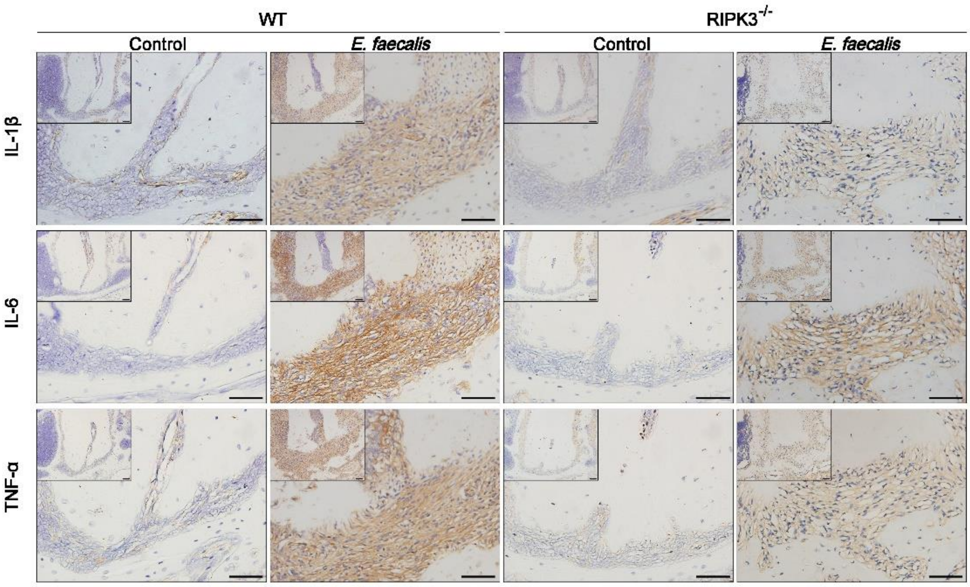

Figure S2 Knockout of *RIPK3* reduces inflammation in an *Enterococcus faecalis*-infected refractory apical periodontitis mouse model. Representative images of immunohistochemical staining of IL-1β, IL-6, and TNF-α in the apical region of the mandibular first molars in wild-type (WT) and *RIPK3*$^{-/-}$ mice with or without *E. faecalis* infection (n = 6/group) (200× and 400× magnification). Scale bar: 50 μm.

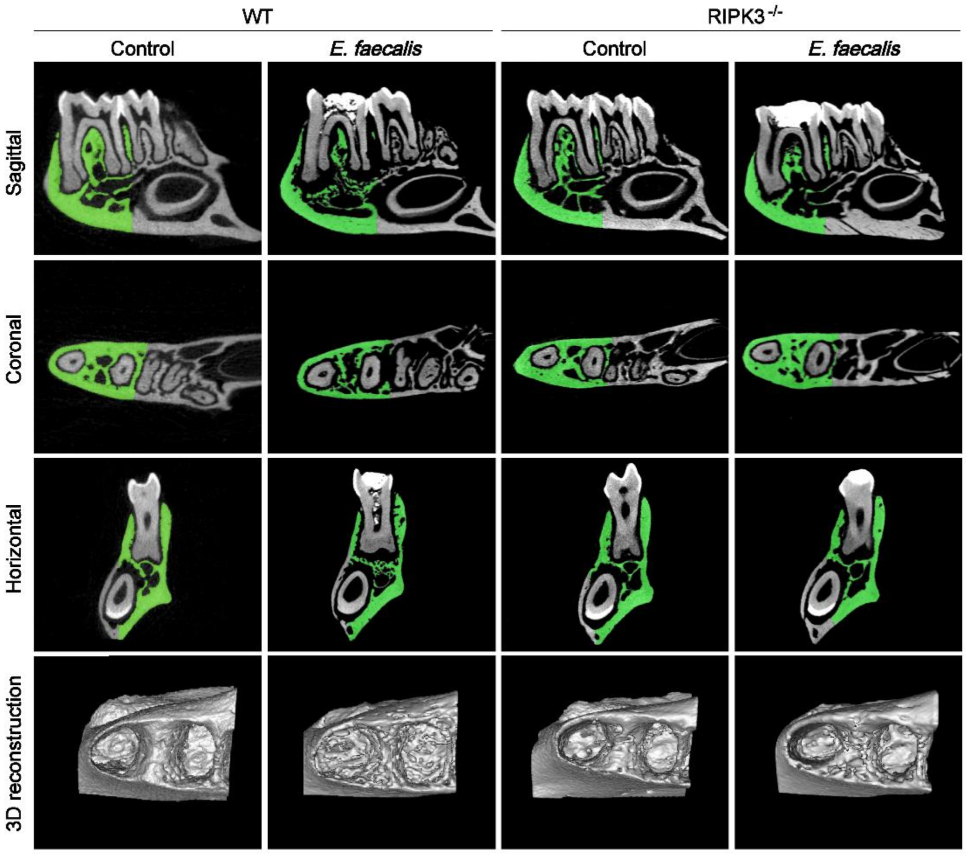

Figure S3 Region of interest selection of residual alveolar bone in Micro-CT analysis. The sagittal, coronal and horizontal view, as well as 3D reconstruction of the residual alveolar bone region of interest of the mandibular first molars in mice.

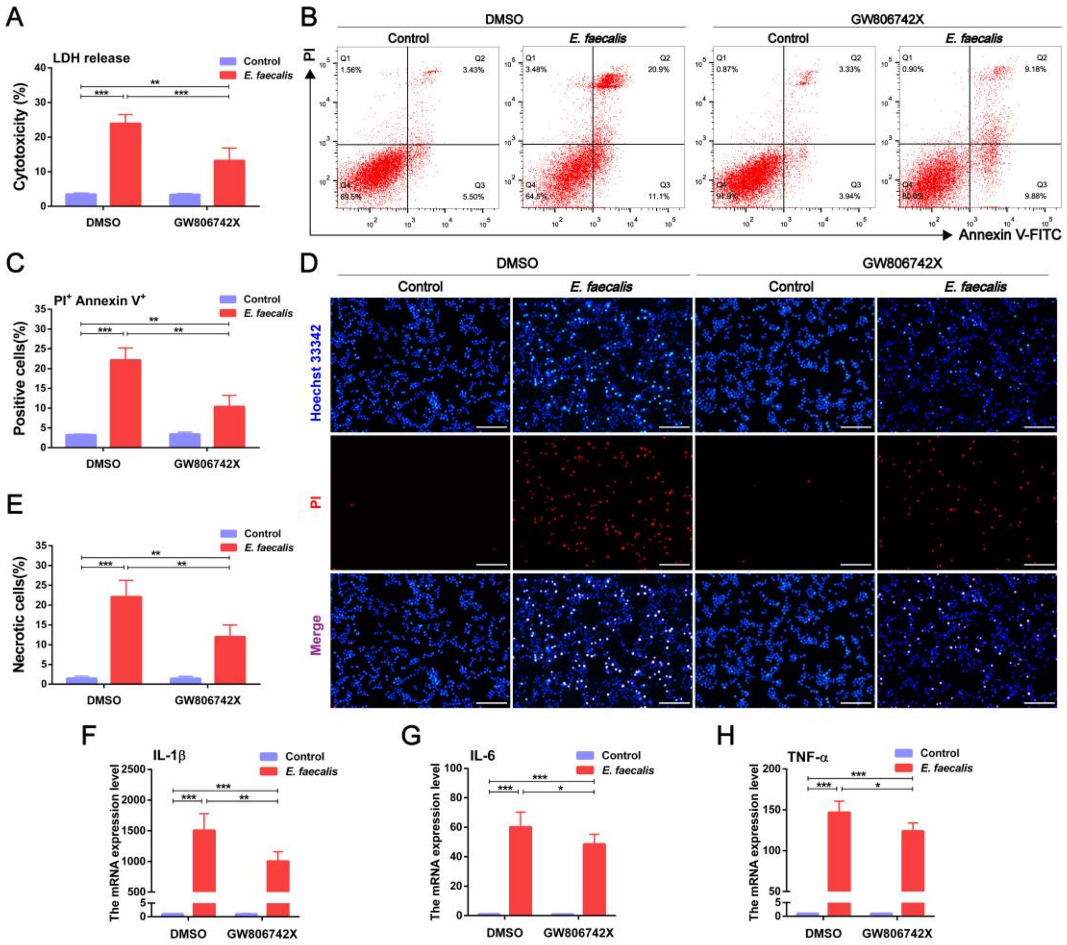

Figure S4 Pretreatment with MLKL inhibitor reduces cell death and inflammation during *Enterococcus faecalis* infection. (A) Lactate dehydrogenase release analysis of *E. faecalis*- infected RAW264.7 cells with an MOI of 100 for 6 h in the presence or absence of GW806742X, the MLKL inhibitor. (B, C) Flow cytometric analysis of propidium iodide and annexin V staining of cells (B) and the quantification of cells double-positive for PI and annexin V staining (C). (D, E) Representative fluorescence images of Hoechst 33342 (blue) and PI (red) double-staining (D) and quantitative analysis of necrotic cells (E) (200× magnification). Scale bar: 100 μm. (F–H) The mRNA expression levels of inflammatory cytokines, including IL-1β (F), IL-6 (G), and TNF-α (H). Results are shown as mean ± SD from three replicates from three independent experiments. Statistical significance was determined using One-way ANOVA with the LSD post hoc test. $^{*}P < 0.05$, $^{**}P < 0.01$, $^{***}P < 0.001$.

Table S1 Primers for RT-qPCR used in this study.

Gene	Species	Sequence (5' to 3')
IL-1β	mouse	F: TGGAGAGTGTGGATCCCAAG
		R: GGTGCTGATGTACCAGTTGG
IL-6	mouse	F: TAGTCCTTCCTACCCCAATTTCC
		R: TTGGTCCTTAGCCACTCCTTC
TNF-α	mouse	F: CAGGCGGTGCCTATGTCTC
		R: CGATCACCCCGAAGTTCAGTAG
β-actin	mouse	F: GCAGGAGTACGATGAGTCCG
		R: ACGCAGCTCAGTAACAGTCC
